# Hydrogen Permeation in X65 Steel under Cyclic Loading

**DOI:** 10.3390/ma13102309

**Published:** 2020-05-17

**Authors:** Marina Cabrini, Luigi Coppola, Sergio Lorenzi, Cristian Testa, Francesco Carugo, Diego Pesenti Bucella, Tommaso Pastore

**Affiliations:** 1 Department of Engineering and Applied Sciences, University of Bergamo, 24129 Bergamo, Italy; marina.cabrini@unibg.it (M.C.); luigi.coppola@unibg.it (L.C.); cristian.testa@unibg.it (C.T.); francesco.carugo@unibg.it (F.C.); diego.pesentibucella@unibg.it (D.P.B.); tommaso.pastore@unibg.it (T.P.); 2 CSGI Consortium, Unity of Bergamo, 24129 Bergamo, Italy; 3 INSTM Consortium, Unity of Bergamo, 24129 Bergamo, Italy

**Keywords:** low-alloyed steel, cathodic protection, hydrogen permeation, elasto–plastic deformation

## Abstract

This experimental work analyzes the hydrogen embrittlement mechanism in quenched and tempered low-alloyed steels. Experimental tests were performed to study hydrogen diffusion under applied cyclic loading. The permeation curves were fitted by considering literature models in order to evaluate the role of trapping—both reversible and irreversible—on the diffusion mechanism. Under loading conditions, a marked shift to the right of the permeation curves was noticed mainly at values exceeding the tensile yield stress. In the presence of a relevant plastic strain, the curve changes due to the presence of irreversible traps, which efficiently subtract diffusible atomic hydrogen. A significant reduction in the apparent diffusion coefficient and a considerable increase in the number of traps were noticed as the maximum load exceeded the yield strength. Cyclic loading at a tensile stress slightly higher than the yield strength of the material increases the hydrogen entrapment phenomena. The tensile stress causes a marked and instant reduction in the concentration of mobile hydrogen within the metal lattice from 55% of the yield strength, and it increases significantly in the plastic field.

## 1. Introduction

On offshore structures, cathodic polarization is usually applied to prevent their generalized corrosion, to reduce the overall loss of metal due to anodic dissolution, and to enhance their corrosion-fatigue limits. However, a correct cathodic protection design is necessary to avoid hydrogen embrittlement (HE) risk [1,2]. Problems can arise on the welded legs of a platform, risers, and on the free span of sea-lines, which are exposed to lateral marine currents; these induce oscillation by vortex shedding [3,4]. Corrosion-fatigue in seawater becomes evident at a frequency below 10 Hz [5,6]. Very high crack growth rates are observed below 1 Hz, at intermediate values of the stress intensity factor range (∆K) and under high stress ratio-R (i.e., high levels of stress) [7]. Cathodic protection plays a positive role in crack initiation, in the absence of propagating defects, by preventing localized attacks [8], but it can increase the fatigue crack growth by promoting atomic hydrogen formation on the surface of the metal [9,10]. Cathodic overprotection (E < −1100 mV vs. SCE) is particularly deleterious for the insurgence of HE phenomena, as reported in the most widespread design guidelines [11,12,13]. It is expected that there would be more evident research on high-strength steels, because they are generally considered more susceptible to hydrogen embrittlement [14,15,16].

Hydrogen assisted cracking has been extensively studied during the last 50 years, but the mechanism is not yet completely known. Several studies formulated a certain number of theories that are all supported by extensive datasets [17,18,19]. One of the most accepted theories is that of pressure, for which the cracking can be ascribed to the recombination of atomic hydrogen into defects. The pressure due to molecular hydrogen recombination promotes void growth and crack propagation as a consequence. In hydride-forming materials, the formation and fracture of brittle hydrides at crack tips trigger hydrogen assisted cracking. Among all the hydrides, niobium, vanadium, anzirconium have been considered [19]. Another theory named surface energy theory postulated that hydrogen absorption decreases the energy of the fracture surface during crack propagation, thus lowering the work of fracture [20]. The hydrogen enhanced decohesion (HEDE) mechanism—also known as hydrogen induced decohesion (HID) mechanism—states that hydrogen concentration at the crack tip lowers the cohesive energy of the lattice, thus decreasing the fracture toughness [21,22,23]. Brittle fractures due to hydrogen embrittlement are explained by these last two mechanisms. Conversely, the hydrogen enhanced localized plasticity theory (HELP) studies the effect of hydrogen on ductile fracture [24,25,26,27]. According to HELP, the dislocation movement promoted by the shear stress decreases, and material softening occurs because of hydrogen redistribution around dislocations. This leads to the reduction in the dislocation’s elastic interaction energy [28]. The onset of nanohydride formation was proposed by Leyson et al. as an additional mechanism associated with HELP [29]. The formation of P, As, Sb, S, Se, and Te hydrides enhances the entry of hydrogen into the steel [30]. The primary function of hydrogen is to enhance the strain-induced nucleation and agglomeration of vacancies according to the hydrogen enhanced strain-induced vacancies (HESIV) theory [31]. Nucleation and the linking of microvoids cause brittle fracture occurrence. An application of different theories or a combined approach (HELP, HEDE, and HESIV) is required to explain the complex phenomena of hydrogen interaction with low-strength steels [32]. 

Pipeline steels having less than 80 ksi tensile strength are not susceptible to HE, as demonstrated by the prolonged service life. HE failures occurred mainly due to the presence of particular microstructures caused by microstructural modifications—i.e., hard spots [33]. Few cases were reported in buried pipelines under slow plastic deformation conditions [34], due to mechanical damages or landslides [35]. Despite several studies having been reported [36,37,38,39,40,41,42,43,44,45,46,47], the HE of pipeline steels is a phenomenon that is far to be completely understood. 

Hydrogen supply at the crack tip has to be granted to support crack propagation, and thus its growth rate is mainly governed by the hydrogen transport rate, the applied stress, and steel intrinsic susceptibility [19,48,49,50]. In any case, there is a general agreement that hydrogen must be continuously supplied and the hydrogen atoms are mainly transported by hydrostatic stresses until critical accumulation occurs at the crack tip [20,51,52,53]. 

Hydrogen diffusion inside different steel microstructures has been widely studied worldwide [54]. Hydrogen flux is proportional to the difference in concentration between surface and metal lattice. The surface concentration increases at very low polarizations, well below protection potentials, and in the presence of recombination poisons. Among all the experimental methods available to measure hydrogen diffusion [55,56,57,58,59,60], the most widespread is surely the one proposed by Devanathan and Stachurski [51,61]. Based on their work, modifications have been proposed for considering the effect of the traps and the kinetics of hydrogen reduction on the cathode surface [55,56,57,58,59,60,62].

The apparent diffusion coefficients in steels at room temperature are extremely scattered. The values lay in the range between 0.1 and 40 × 10^−10^ m^2^s^−1^, but they are spread in the range between 1 and 5 × 10^−10^ m^2^s^−1^ [63].

Several parameters influence hydrogen diffusion inside metals—first, the steel microstructure, and then the presence of second phases [64]. On the other hand, a strong effect of an applied loading is generally accepted [6]. 

In previous studies, a correlation was found between the hydrogen diffusion coefficient and the hydrogen embrittlement index in either slow strain rate tests or on the crack growth in corrosion-fatigue tests, using a hydrogen diffusion coefficient evaluated in the absence of an external load [7,65]. Other studies evaluated hydrogen diffusion under static load conditions and slow strain rate conditions [66,67,68,69].

In this paper, hydrogen diffusion under applied cyclic loading on sorbitic X65 grade steel is studied. Permeation transients are analyzed by considering literature models in order to evaluate the role of trapping—both reversible and irreversible—on the diffusion mechanism during the application of load in the elastic and plastic field. In addition, the effect of the application of different cyclic loading conditions on steady state hydrogen permeation flux is analyzed. 

Despite the topic having already attracted the attention of several researchers, the paper would increase the knowledge of the effect of cyclic loading on hydrogen diffusion to give more insights into the topics related to hydrogen embrittlement of high-strength low-alloyed steel. 

## 2. Experimental Section

**Figure 1:**
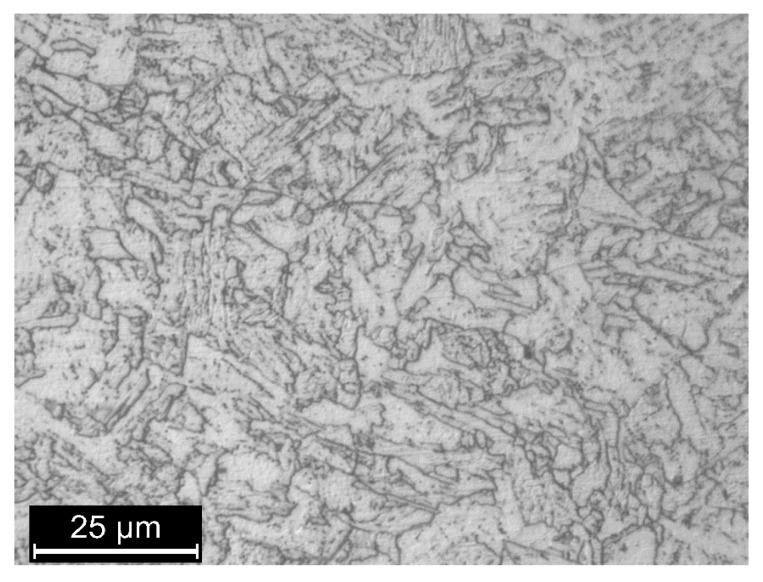
Microstructure of X65 grade steel after metallographic etching (Nital 2%).

Commercial pipeline steel (X65 grade according to API 5L standard) having a sorbitic microstructure (Figure 1) [70] was considered during this experimentation. The microstructure was revealed after polishing with emery papers up to 1200 grit and degreased with acetone in an ultrasonic bath.

**Figure 2:**
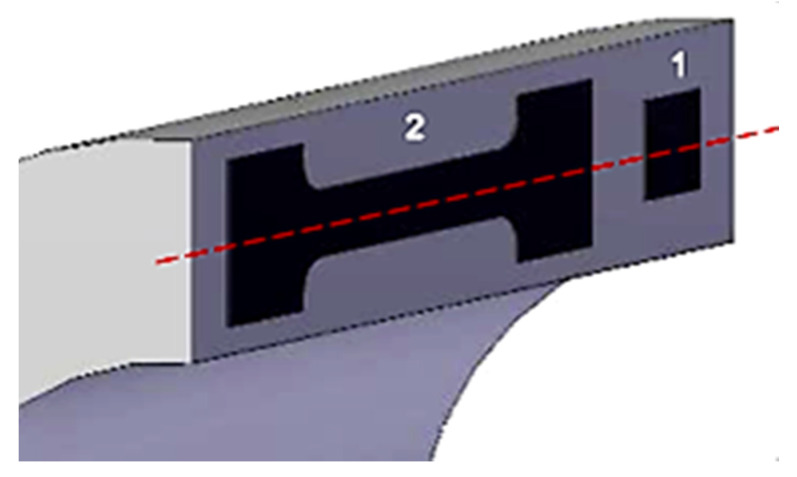
Specimens for the permeation tests: (1) In the absence of load; (2) Under cyclic loading.

The specimens were taken from a commercial thick wall seamless pipe (outer diameter equal to 360 mm, thickness equal to 57 mm). They were obtained from the center of the pipe thickness (Figure 2) to ensure that hydrogen permeation occurrence is always at the same depth. Hydrogen permeation tests were carried out on unloaded specimens (U, rectangular sheet, Figure 2; Specimen 1) and dog-bone specimens for cyclic loading tests (C, Figure 2; Specimen 2 according to EN 10002-1 standard).

Specimens were cut by using a cutting machine for metallographic sample preparation to avoid the occurrence of residual stresses inside the material. The specimens were then milled to a uniform thickness of 1 ± 0.01 mm at the exposed area. The thickness was measured by means of Mitutoyo’s High-Accuracy Digimatic Micrometer (Kawasaki, Japan). Before the tests, the specimens were grounded with emery paper and polished with diamond paste of up to 1 µm finishing.

Tensile strength was measured on specimens with the same dimensions as those used in all the permeation tests under cyclic loading conditions. The tensile tests, performed on an oleo-dynamic universal testing device, showed values of the tensile yield strength (TYS) and the ultimate tensile strength (UTS) equal to 450 and 570 MPa, respectively. The different loading levels in the cyclic loading tests were then determined according to these two values.

A Devanathan–Stachurski cell was used. The layout of the cell was modified for the tests under cyclic loading. Poly-methyl-methacrylate (PMMA) compartments and flanges opportunely designed to hold the specimen by means of flat neoprene gaskets were applied as sealers. The exposed area in both the charging side (cathodic compartment) and detection side (anodic compartment) was 1.76 cm^2^, considering the neoprene deformation after the cell assembly. The ratio between the radius and thickness of the membrane was greater than 5:1, as recommended by [61]. Both the charging and detection side solutions were thermally regulated at a constant temperature of 23 ± 0.5 °C, as the results can be significantly affected by temperature variations.

A sodium hydroxide 0.1 M aerated solution was considered in the detection side, in stagnant conditions. A potentiostatic anodic polarization of +340 mV vs. Ag/AgCl (3 M KCl) was applied by using an AMEL General Purpose Potentiostat/Galvanostat (Model 2049, Milan, Italy). The anodic surface was not Pd-coated because rupture of this electrodeposited thin Pd layer is expected under loading deformation [71], even in the elastic field. An MMO-activated titanium net was used as a counter electrode in both the compartments. Once anodic current densities lower than 0.05 µA/cm^2^ were recorded—well below the limit of 0.1 µA/cm^2^ suggested by [61]—hydrogen charging was activated in the charging side of the cell. The cathodic surface of the specimen was then polarized at constant current density.

An aerated borate solution (0.3 M H_3_BO_3_ + 0.075 M Na_2_B_4_O_7_) buffered at pH 8.4 [72] was inserted in the charging compartment. As stated by Nagumo [73], the borate buffer solution pH is stable during long-term testing. An immersion pump guaranteed solution recirculation in the cathodic compartment. The flow rate was fixed at values around 1 m/s to further minimize alkalization effects.

The anodic current density in the detection compartment was measured with a frequency of 1 Hz to synchronize the applied load with the resulting current signal.

The loading sinusoid was characterized by a maximum value in the range of 55–120% TYS, an amplitude equal to ±10–20% TYS, and a frequency from 1 to 10^−2^ Hz. The tests were summarized as follows.

- A first series of tests was performed by applying the cyclic load before the activation of the charging cathodic current, and then it was maintained simultaneously to hydrogen permeation transient in order to evaluate the effect of cyclic loading on the apparent hydrogen permeation coefficient (Table 1).
- A second series of tests was conducted by applying the cyclic load after the achievement of the steady state of hydrogen permeation to evidence its effect on the hydrogen flux at equilibrium. These tests were also performed in the absence of a hydrogen charge to evaluate the effect of the cyclic load on the passivity current density (Table 2).
- A third series of tests was performed in order to evaluate the transient of the hydrogen steady state flux and of the passive current, increasing on the specimen the mean value of the applied load from 0% to 110% TYS, maintaining constant the amplitude and the frequency (Table 3).
- Some tests were carried out on a specimen subject to the cyclic loading for the first time from 0% to 55% TYS and from 55% to 90% TYS, then unloaded and loaded again from 0% to 90% TYS.

The experiments were almost twice conducted, and the results showed a good reproducibility.

The data were processed with a method based on the mathematical model proposed by Grabke and Riecke [74], which takes into consideration the method implemented by Oriani [75] and integrates it by considering the presence of both reversible (shallow) traps and irreversible (deep or strong) traps.

## 3. Results and Discussion

**Figure 3:**
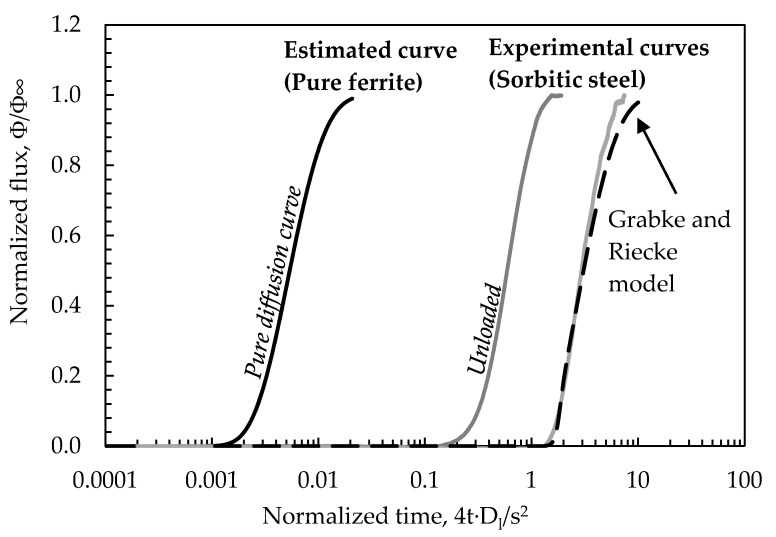
Experimental permeation curves as adimensional flux/adimensional time (Φ = hydrogen flux; Φ_∞_ = steady state flux; t = permeation time; s = thickness): Comparison between the modeled permeation curve and the experimental data curve.

Figure 3 shows the effect of the cyclic loading on permeation flux. The model proposed by Grabke and Riecke [74,76] was applied for fitting the transient data. More detailed information on data processing is reported in [64]. Based on this model, the effect of reversible traps can be described as a shift of the time needed to reach the steady state value. The effect of irreversible traps can be mainly noticed by analyzing the slope of the curve.

**Figure 4:**
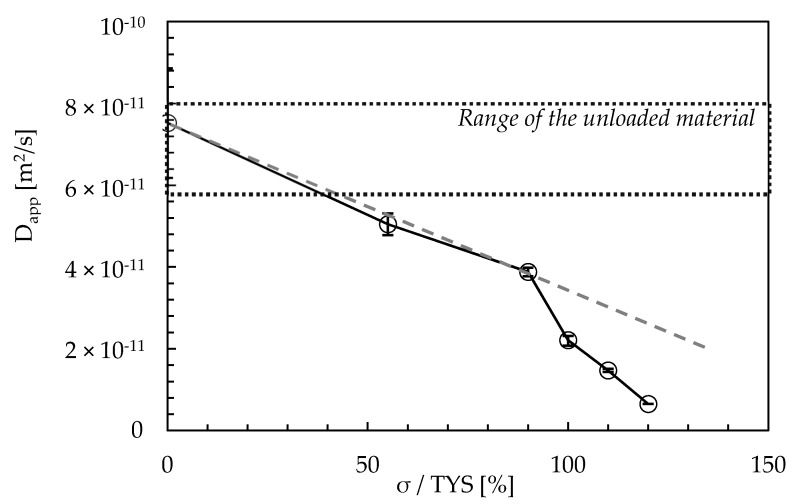
Relation between *D*_app_ (apparent diffusivity) and applied stress.

The hydrogen permeation transient was expressed as a function of cyclic loading parameters. The relation between apparent diffusivity (D_app_) and cyclic loading conditions is shown in Figure 4. Data are compared to the unloaded condition as a function of the ratio between the applied nominal stress (σ) and the TYS value.

D_app_ decreases with the maximum tensile stress, with values in the range between 1.5 × 10^−11^ and 6.5 × 10^−12^ m^2^/s. The decrease in D_app_ as the applied maximum stress increases can be mainly related to the growth in the dislocation density [76,77,78]. The decrease in D_app_ can already be noticed in the elastic field (55% TYS), although to a lesser extent. This becomes much more evident when exceeding the stress at yield. The sudden decrease in D_app_ values can then be ascribed to the creation of a large quantity of new reversible traps, i.e., new dislocations [79].

For the considered loading conditions, plastic deformation determines a significant increase in the trap density, which reduces the diffusible hydrogen in the lattice and causes a reduction in the hydrogen flux during the transient [80].

**Figure 5:**
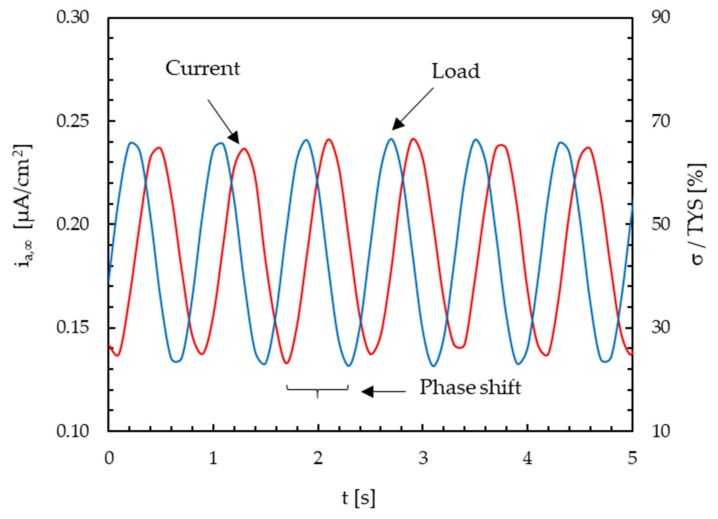
Steady state anodic current (amplitude = ±20% tensile yield strength (TYS), frequency = 1 Hz) in function of an alternate component of loading.

The effect of an alternate component of loading at ±120% TYS (frequency = 1 Hz) and the steady state current is shown in Figure 5. The current signal is modified similarly to the applied load. The same behavior can be detected for passive current density in the absence of hydrogen. Fast Fourier transform (FFT) and inverse fast Fourier transform (IFFT) were then applied to the sinusoidal signals to obtain the average value and amplitude of i_a,∞_ and i_P,∞_ for each value of the amplitude and frequency of the load.

**Figure 6:**
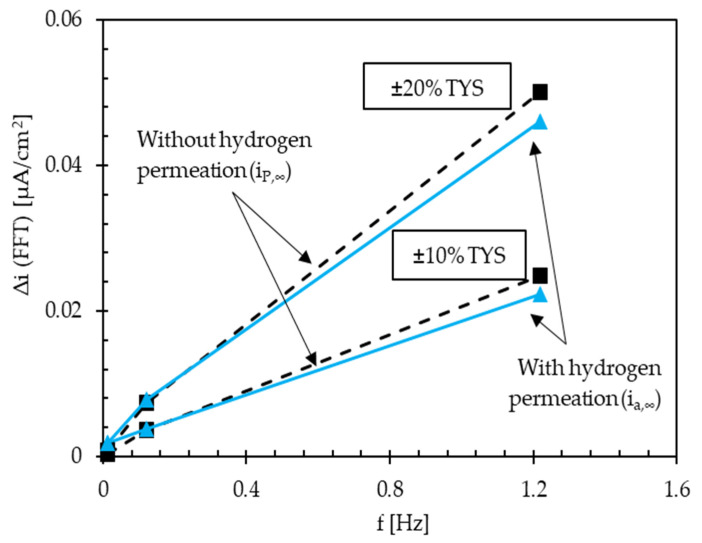
Current variation as a function of load amplitude and frequency, and comparison between the cases of the presence and absence of diffusible atomic hydrogen.

Figure 6 reports the increase in the current response (Δi (FFT)) both for i_a,∞_ and i_P,∞_, in function of the load amplitude (Δσ) and frequency (f). The current variation induced by loading (amplitudes: ±10% and ±20% TYS; significant frequencies: 10^−2^, 10^−1^, 1 Hz for each load amplitude) on i_a,∞_ in the presence of hydrogen is slightly lower than the variation induced on i_P,∞_ in the absence of hydrogen.

The variation in the passive current density with the cyclic loading increases with the frequency. This effect could be ascribed to the repassivation of new exposed metal surfaces due to the loading and the variation of the internal energy of the oxide film in the elastic field. The cyclic loading could slightly deplete the metal lattice of hydrogen atoms, which are trapped in shallow traps, with the result of a decrease in the permeation flux during the cyclic loading [44]. This grows with the increase in the passive current, and the result is a growth of the anodic current variation lower than that observed in the absence of hydrogen.

**Figure 7:**
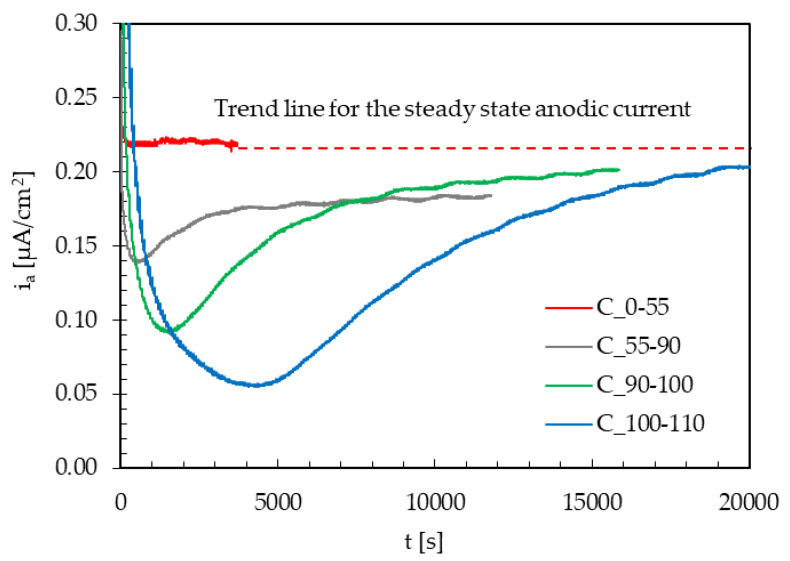
Effect of instant variations of the maximum load (amplitude = ±10% TYS; frequency = 10^−2^ Hz) on the steady state anodic current (i_a,∞_).

The effect of the instant variations of the maximum load under cyclic loading conditions on i_a,∞_ in the presence of hydrogen was evaluated with some tests performed at the lowest frequency (10^−2^ Hz) and with an amplitude of the cyclic load equal to ±10% TYS (Figure 7). All the permeation transients are obtained from the same specimen, starting from the unloaded condition and then increasing the average value of loading.

As a title of the example, the curve indicated as “C_100-110” refers to a variation in the applied maximum stress from 100% to 110% TYS. The loading variation was applied consequently once i_a,∞_ was achieved under cyclic loading conditions at an applied maximum stress of 100% TYS.

For the loading variation from 0% to 55% TYS, no appreciable variations in the hydrogen flux can be noticed. As loading variations approached the yield limit (from 55% to 90% TYS and from 90% to 100% TYS) and exceeding that limit (from 100% to 110% TYS), an instantaneous peak in the current at the loading variation is detected, and a marked drop to values well below the initial steady state flux is seen. Then, a transient can be evidenced, and the current slowly returns to values approaching the steady state reached prior to the loading variation.

**Figure 8:**
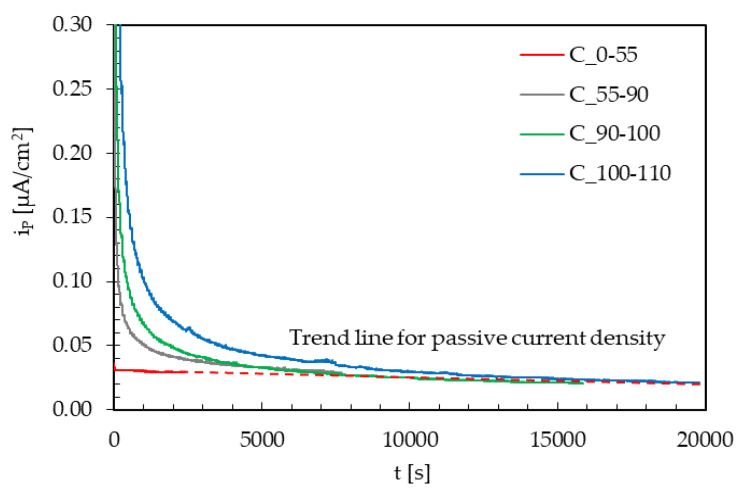
Effect of instant variations of the maximum load (amplitude = ±10% TYS; frequency = 10^−2^ Hz) on the background passivity current (i_P,∞_).

However, when the same loading variations were applied on specimens without hydrogen charging—i.e., on the i_P,∞_ (Figure 8)—again on the same specimen and continuously, the instantaneous peak is always present at the loading variation. This suggests that this phenomenon is due to the instant local rupture of the oxide film and not to a variation in the hydrogen flux.

Therefore, after the rupture of the oxide film, repassivation takes place along with a temporary decrease in the permeation flux, due to the creation of new trapping sites that temporarily reduce the concentration of diffusible hydrogen in the metal lattice. This effect seems to be quite negligible for loading variations in the elastic field. It becomes even higher just approaching the yield limit, and it further increases well exceeding this value.

**Figure 9:**
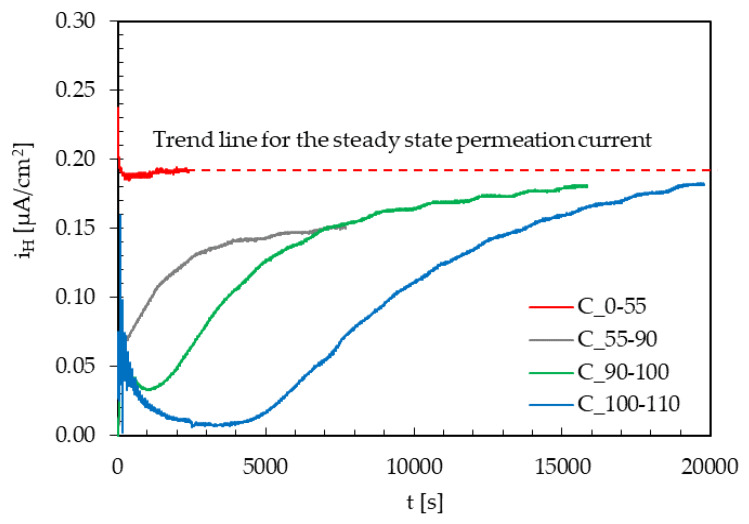
Effect of instant variations of the maximum load (amplitude = ±10% TYS; frequency = 10^−2^ Hz) on the steady state hydrogen permeation current (i_H,∞_).

In order to discriminate hydrogen trapping effects, the value of i_a,∞_ (Figure 9) has to be reduced to take into account the increasing value of i_P,∞_ as loading increases due to this activation–repassivation mechanism occurring at steel surface.

The effect of the decrease in diffusible hydrogen concentration in the lattice is due to the generation of new traps by the plastic deformation; this phenomenon is also present in the elastic field, and it increases as the applied loading increases, as expected. Once the new generated traps are filled, the steady state current returns to the initial value.

**Figure 10:**
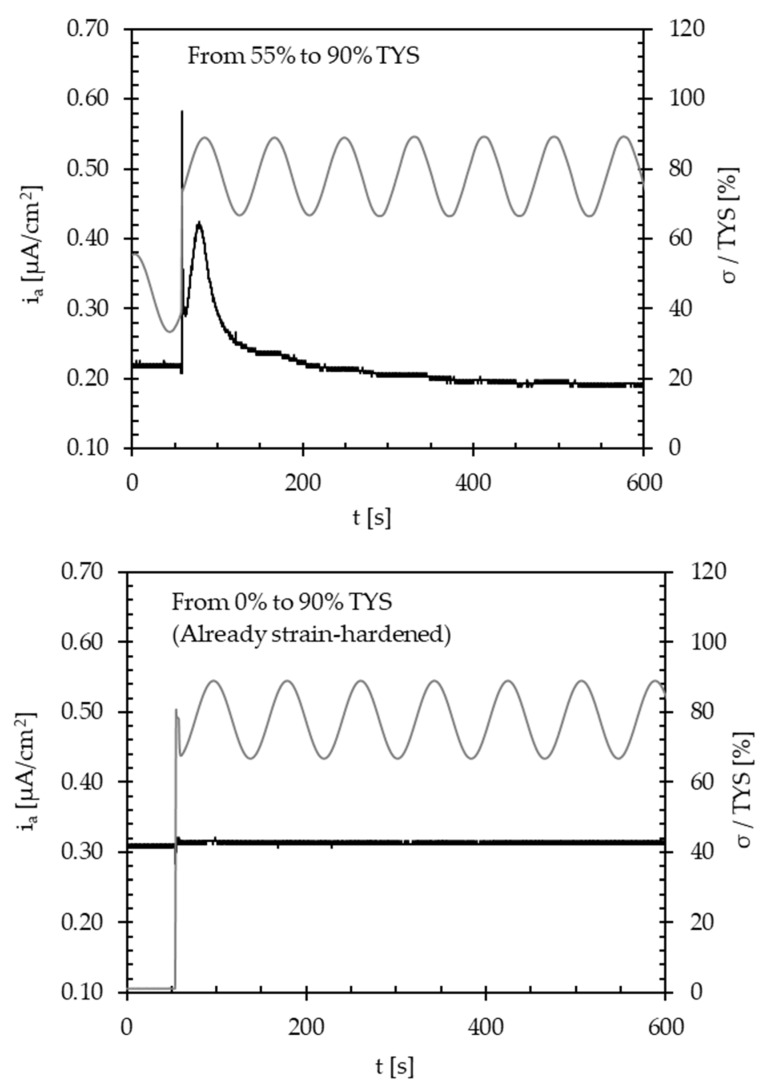
Response to subsequent variations of the maximum load (amplitude = ±10% TYS; frequency = 10^−2^ Hz) on the steady state anodic current (locally strain-hardened steel).

Some tests were carried out on specimens subject to the cyclic loading for the first time, which were then unloaded and loaded again with the same cyclic load applied before (Figure 10) to support this hypothesis. As a result, no transient decrease in the permeation flux was detected in this case, probably due to the local strain-hardening of the material and no creation of new trapping sites.

A decrease in the hydrogen stationary flux by only applying the external load was observed in previous works of the same authors under constant load [7], according to other results also [69]. In constant strain rates conditions [7,81,82,83], the hydrogen permeation flux decreases with the strain rate. At very high strain rates, decrease in the hydrogen permeation flux can be noticed as plastic deformation increases due to the filling of new traps, thus causing a decrease in the hydrogen concentration inside the metal lattice. At strain rates below 10^−6^ s^−1^, the rate of trap generation is lower than the saturation rate, and hence depletion cannot occur; the trapping effect becomes negligible with respect to diffusion. The hydrogen concentration in plastically strained zones was well evidenced by means of photoelectrochemical techniques [84].

The embrittlement effect evaluated by means of slow strain rate tests and crack growth rate tests under corrosion-fatigue conditions can be related to the hydrogen apparent diffusion coefficient only for steel having similar microstructures [24,25,84,85]. The hydrogen embrittlement effects were evaluated by considering the presence of brittle areas on the fracture surfaces. No evidence of HELP was observed as the stress–strain curves of the specimens with ductile fracture were always similar than those at air. The decreases in the total elongation at break and in the reduction of area were always observed once brittle initiation of the fracture occurred. In many cases, it was always noticed that the role of plastic strain was relevant as HE was always initiated at the necking. Crack propagation was never observed to occur in absence of dynamic loading, i.e., under constant loading conditions. In this work, the continuous straining in the plastic field occurs mainly during the increasing of loading in the first sinusoidal waveform. The effects of local straining are evident in the hydrogen permeation transient once the specimen is charged with hydrogen after loading. On the contrary, the load acts mainly on the stationary value of the hydrogen permeation flux, which modifies as a function of the frequency. At very low loading frequencies, it can be assumed that the loading conditions are like those under a monoaxial slow strain rate. The trapping effect becomes prevalent at high frequencies and decreases at low frequencies.

## 4. Conclusions

The results underline the role of cyclic loading on hydrogen transport mechanisms in commercial API 5L X65 steel. The following conclusions can be drawn:

- The apparent diffusion coefficient (D_app_) ranges between 6.2 × 10^−11^ and 8.9 × 10^−11^ m^2^/s, with an average value of 7.5 × 10^−11^ m^2^/s. Under cyclic loading conditions, the application of a tensile stress up to yield determines a slight decrease in D_app_. When exceeding such a value—i.e., at 110% TYS—D_app_ decreases to values around 1.4 × 10^−11^ m^2^/s due to the creation of a large quantity of new reversible and irreversible trapping sites.
- An applied cyclic load causes a very small decrease in the hydrogen permeation current at the steady state, mainly hindered by the increase in the passive current. This is slight as the cycle frequency decreases, and it significantly increases as the load amplitude increases.
- Increase in the maximum load causes a temporary decrease in the permeation flux, which becomes larger and larger as the applied load increases. Once the new traps generated are saturated with diffusible hydrogen, the steady state current returns to the value prior to load increase.
- No transient modifications in the permeation flux are detected after subsequent load variations on the specimens already subject to the same load variation (already strain-hardened), probably because of the local strain-hardening of the material and no creation of new trapping sites.

## Figures and Tables

**Table 1:** Testing conditions of the tests on apparent hydrogen diffusion coefficient.

Label	Maximum Load	Amplitude	Frequency	i_C_ (mA/cm^2^)
C_55	55% TYS	±10% TYS	1.22 × 10^−2^ Hz	−0.50
C_90	90% TYS	±10% TYS	1.22 × 10^−2^ Hz	−0.50
C_100	100% TYS	±10% TYS	1.22 × 10^−2^ Hz	−0.50
C_110	110% TYS	±10% TYS	1.22 × 10^−2^ Hz	−0.50

**Table 2:** Test conditions for the evaluation of the effect of cyclic loading on the hydrogen stationary permeation flux and the passivity current.

Label	Maximum Load	Amplitude	Frequency	i_C_ (mA/cm^2^)
C_45_10_f1_H	45% TYS	±10% TYS	1.22 × 10^−2^ Hz	−0.50
C_45_10_f2_H	45% TYS	±10% TYS	1.22 × 10^−1^ Hz	−0.50
C_45_10_f3_H	45% TYS	−10% TYS	1.22 Hz	−0.50
C_45_20_f1_H	45% TYS	±20% TYS	1.22 × 10^−2^ Hz	−0.50
C_45_20_f2_H	45% TYS	±20% TYS	1.22 × 10^−1^ Hz	−0.50
C_45_20_f3_H	45% TYS	±20% TYS	1.22 Hz	−0.50
C_45_10_f1	45% TYS	±10% TYS	1.22 × 10^−2^ Hz	0
C_45_10_f2	45% TYS	±10% TYS	1.22 × 10^−1^ Hz	0
C_45_10_f3	45% TYS	±10% TYS	1.22 Hz	0
C_45_20_f1	45% TYS	±20% TYS	1.22 × 10^−2^ Hz	0
C_45_20_f2	45% TYS	±20% TYS	1.22 × 10^−1^ Hz	0
C_45_20_f3	45% TYS	±20% TYS	1.22 Hz	0

**Table 3:** Test conditions for the evaluation of the effect of the increasing of the mean load on the hydrogen stationary permeation flux and the passivity current.

Label	Increasing Od Mean Load	Amplitude	Frequency	i_C_ (mA/cm^2^)
C_0_55	From 0% to 55% TYS	±10% TYS	1.22 × 10^−2^ Hz	−0.50
C_55_90	From 55% to 90% TYS	±10% TYS	1.22 × 10^−2^ Hz	−0.50
C_90_100	From 90% to 100% TYS	±10% TYS	1.22 × 10^−2^ Hz	−0.50
C_100_110	From 100% to 110% TYS	±10% TYS	1.22 × 10^−2^ Hz	−0.50
